# Magnetite Nanoparticles Induce Genotoxicity in the Lungs of Mice via Inflammatory Response

**DOI:** 10.3390/nano4010175

**Published:** 2014-03-18

**Authors:** Yukari Totsuka, Kousuke Ishino, Tatsuya Kato, Sumio Goto, Yukie Tada, Dai Nakae, Masatoshi Watanabe, Keiji Wakabayashi

**Affiliations:** 1 Division of Cancer Development System, National Cancer Center Research Institute, 1-1 Tsukiji 5-chome, Chuo-ku, Tokyo 104-0045, Japan; E-Mails: ytotsuka@ncc.go.jp (Y.T.); kishino@nms.ac.jp (K.I.); katou.tatsuya@ma.mt-pharma.co.jp (T.K.); 2 Laboratory of Environmental Risk Evaluation, School of Life and Environmental Science, Azabu University, 1-17-71 Fuchinobe, Chuou-ku, Sagamihara, Kanagawa 252-5201, Japan; E-Mail: gotou@azabu-u.ac.jp; 3 Department of Pharmaceutical and Environmental Sciences, Tokyo Metropolitan Institute of Public Health, 3-24-1 Hyakunin-cho, Shinjuku-ku, Tokyo 169-0073, Japan; E-Mails: yukie_tada@member.metro.tokyo.jp (Y.T.); agalennde.dai@nifty.com (D.N.); 4 Department of Food and Nutritional Science, Tokyo University of Agriculture, 1-1-1 Sakuragaoka, Setagaya-ku, Tokyo 156-8502, Japan; 5 Division of Materials Science and Engineering, Graduate School of Engineering, Yokohama National University, Hodogaya-ku, Yokohama 240-8501, Japan; E-Mail: mawata@ynu.ac.jp; 6 Graduate Division of Nutritional and Environmental Sciences, University of Shizuoka, 52-1, Yada, Shizuoka 422-8526, Japan

**Keywords:** magnetite nanoparticle (MGT), pulmonary inflammation, intratracheal instillation, DNA damage, genotoxicity

## Abstract

Nanomaterials are useful for their characteristic properties and are commonly used in various fields. Nanosized-magnetite (MGT) is widely utilized in medicinal and industrial fields, whereas their toxicological properties are not well documented. A safety assessment is thus urgently required for MGT, and genotoxicity is one of the most serious concerns. In the present study, we examined genotoxic effects of MGT using mice and revealed that DNA damage analyzed by a comet assay in the lungs of imprinting control region (ICR) mice intratracheally instilled with a single dose of 0.05 or 0.2 mg/animal of MGT was approximately two- to three-fold higher than that of vehicle-control animals. Furthermore, in *gpt* delta transgenic mice, *gpt* mutant frequency (MF) in the lungs of the group exposed to four consecutive doses of 0.2 mg MGT was significantly higher than in the control group. Mutation spectrum analysis showed that base substitutions were predominantly induced by MGT, among which G:C to A:T transition and G:C to T:A transversion were the most significant. To clarify the mechanism of mutation caused by MGT, we analyzed the formation of DNA adducts in the lungs of mice exposed to MGT. DNA was extracted from lungs of mice 3, 24, 72 and 168 h after intratracheal instillation of 0.2 mg/body of MGT, and digested enzymatically. 8-Oxo-7,8-dihydro-2′-deoxyguanosine (8-oxodG) and lipid peroxide-related DNA adducts were quantified by stable isotope dilution liquid chromatography-mass spectrometry (LC-MS/MS). Compared with vehicle control, these DNA adduct levels were significantly increased in the MGT-treated mice. In addition to oxidative stress- and inflammation related-DNA adduct formations, inflammatory cell infiltration and focal granulomatous formations were also observed in the lungs of MGT-treated mice. Based on these findings, it is suggested that inflammatory responses are probably involved in the genotoxicity induced by MGT in the lungs of mice.

## 1. Introduction

Magnetite nanoparticles (MGT), a form of iron oxide (Fe_3_O_4_) nanoparticles have been widely exploited since the simplicification of synthesis, mainly because of their unique magnetic properties. Applications include use in printing inks and magnetic recording media [1]. Moreover, MGTs are also used for various fields including medical applications, such as magnetic resonance imaging, hyperthermia, and drug delivery [2,3,4,5,6]. Some reports have demonstrated that MGTs have a weak toxicity compared to other metal oxides, including titanium dioxide [7,8], however, there is still controversy regarding their toxicity. Hitherto, several reports describing MGT toxicity have been published, but most investigations focus on studying effects of MGT on *in vitro* cellular viability, morphology and metabolism, or *in vivo* general toxicity on the various organs using rats/mice by various administration routes (intraperitoneal, intratracheal or intravenous injection) with MGT [7,8,9,10,11,12,13,14,15,16]. For example, exposure of cultured mammalian cells to MGT induces not only cytotoxicity and genotoxicity [10,11,12], but also an inflammatory response and the generation of reactive oxygen species (ROS) [8,11]. We also recently demonstrated that MGT actually leads to oxidative stress, cytotoxicity and micronuclei induction against cultured mammalian cells [17,18]. Similarly, MGT induce cytotoxic and genotoxic effects in lungs of mice [19]. Moreover, it has been demonstrated that after instillation of MGT in mice, an increased expression of pro-inflammatory cytokines, such as IL-6, TNF-α and IL-6, and intracellular reduced glutathione is observed [19]. Expression of inflammation related genes and formation of microgranulomas are two of the indicators for a chronic inflammatory response [19]. Based on this information, it is suggested that MGTs might increase the inflammatory response in both cultured mammalian cells and animals, and then induce toxicity. However, how the inflammatory response contributes to the genotoxic action of MGT has not fully been elucidated up to this point. Moreover, there are no reports that clearly demonstrate mutagenic activity induced by MGT. Therefore, it is necessary to clarify whether MGT is able to induce mutations *in vivo* or not, and what mechanisms are involved in *in vivo* genotoxicity induced by MGT.

Here, we evaluated the *in vivo* genotoxicity, including DNA damage and mutagenicity, of MGT in the lungs of both wild-type and transgenic mice to obtain fundamental data to elucidate the health risk of MGT. Furthermore, aiming at examining the mechanisms involved in genotoxicity induced by MGT, oxidative and lipid peroxide-related DNA adduct analysis was also performed. In the present study, MGT was demonstrated to be genotoxic/mutagenic in all of the *in vivo* tests. Possible mechanisms are also suggested.

## 2. Results

### 2.1. Characterization of MGT

**Figure 1:**
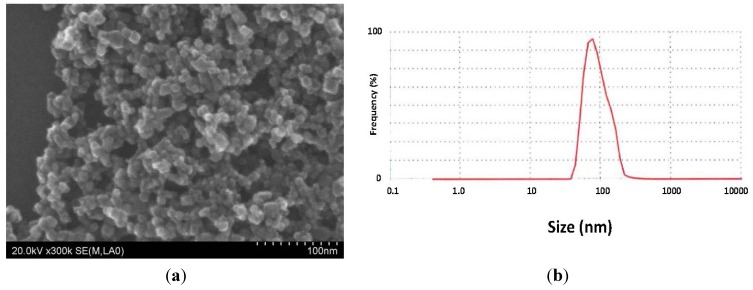
Crystal appearance and zeta potential of magnetite nanoparticles (MGT): (**a**) Scanning electron microscopy (SEM) micrographs of MGT obtained at *E*=20 kV,×300,000;and (**b**) Size distribution of MGT measured in water, 0.2 μg/mL.

In order to characterize and ascertain the properties of MGT used in the present study, the particle appearance, dispersed diameter and zeta potential were determined. Figure 1a shows scanning electron microscopy (SEM) images of MGT. The particles are smoothly sphere-shaped with a mean particle size of 12.5 ± 4.45 nm. Good agreement was indicated between declared primary particle size by the industrial company and particle size from the SEM study. Size distribution in water is shown in Figure 1b. The major peak is around 90 nm (average size 95.19 nm), indicating that MGT particles exist in a partly agglomerated state. Surface charge was determined as zeta potential, and MGT particles showed around −20 mV at pH 7.7.

### 2.2. In Vivo Genotoxicity of MGT

**Figure 2:**
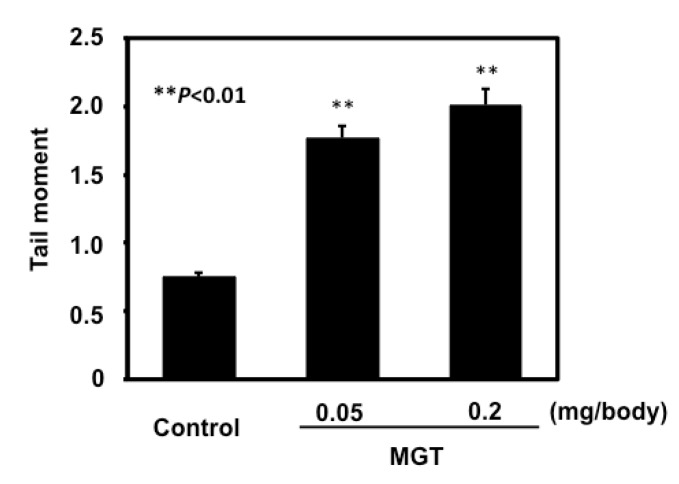
DNA damage in the lungs of imprinting control region (ICR) mice intratracheally instilled with MGT. DNA damage was measured by comet assay. Male micewere treated at a dose of 0.05 mg or 0.2 mg of particles per animal, and sacrificed 3 h after particle administration. The values represent the means of data for five animals ± SE. ** *P* < 0.01, by the Dunnett’s test after one-way analysis of variance *vs*. the corresponding vehicle control mice.

To assess the DNA damage of MGT, we performed a comet assay under alkaline conditions for the lungs of mice intratracheally instilled with MGT. The mean values of DNA tail moment in the lungs with or without a 3 h MGT treatment at 0.05 or 0.2 mg/animal are shown in Figure 2. DNA damage observed in the MGT-treated group was significantly increased in a dose-dependent manner compared with those of the vehicle control.

### 2.3. gpt and Spi^−^ Mutations in the Lungs of gpt Transgenic Mice Treated with MGT

**Figure 3:**
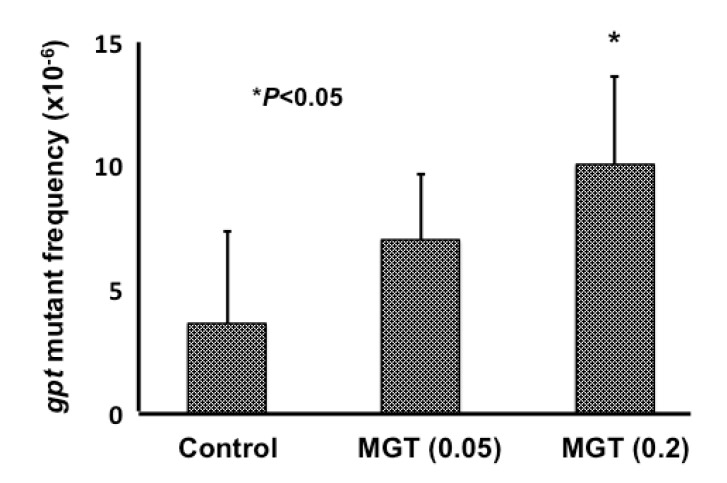
The *gpt* mutation frequencies in the lungs of mice after multiple intratracheal instillations of MGT. Male mice were treated with multiple (0.05 or 0.2 mg/mouse × 4 times) doses of MGT, and mice were sacrificed eight weeks after MGT administration. The data represent the mean ± SD; * *P* < 0.05 by the Student’s *t*-test *vs.* the corresponding vehicle control mice.

All mice in this study behaved normally and survived throughout the experimental period. The treated animals did not show fatigue, loss of appetite or weight loss. Figure 3 shows *gpt* mutant frequencies (MFs) in the lungs of *gpt* delta transgenic mice exposed to four consecutive intratracheal instillations of 0.05 mg or 0.2 mg MGT.

Data are also summarized in Table 1. MF in the lungs of mice treated with low-dose MGT was about two times higher than that of the control group, but the difference is not statistically significant (*P* = 0.09). However, high-dose instillation of MGT resulted in a significant, approximately three-fold increase in the MF compared with that in controls. We also measured the Spi^−^ MFs in the lungs of *gpt* delta mice instilled with compared to those without MGT, but no differences were observed between the control and MGT exposed groups (data not shown).

**Table 1:** Summary of mutant frequency (MF) in the lungs of *gpt* delta mice treated with MGT.

Treatment		Mouse ID	Number of colonies		MF (×10^−6^)	Average MF (×10^−6^) *
			Mutant	Total		
**Control ^†^**		1	6	747,000	8.03	-
		2	2	592,500	3.38	-
		3	14	2,998,500	4.67	-
		4	3	2,937,000	1.02	-
		5	2	1,759,500	1.14	-
		Total	27	9,034,500	-	3.65 ± 3.69
**MGT**	**0.05 (mg/body) × 4**	1	2	621,900	3.22	-
		2	7	903,000	7.75	-
		3	5	666,000	7.51	-
		4	5	814,500	6.14	-
		5	7	669,000	10.46	-
		Total	26	3,674,400	-	7.02 ± 2.64
	**0.2 (mg/body) × 4**	1	2	502,500	3.98	-
		2	5	475,500	10.52	-
		3	5	546,000	9.16	-
		4	12	846,000	14.18	-
		5	5	522,000	9.58	-
		6	1	78,000	12.82	-
		Total	30	2,970,000	-	10.04 ± 3.54 ^‡^

* Mean ± SD; ^†^ Solvent control (treatment with 0.05% (*v*/*v*) Tween 80); and ^‡^ *P* < 0.05 (*vs.* solvent control) by the Student’s *t*-test.

To analyze the spectra of the mutations induced by MGT, we tested for 6-thioguanine (6-TG)-resistant mutants using polymerase chain reaction (PCR) and DNA sequencing analysis. We used 30 independent 6-TG-resistant mutants derived from high-dose MGT instillations, and 27 mutants from vehicle controls. The classes of mutation found in the *gpt* gene are summarized in Table 2. Base substitutions predominated in MGT-induced and spontaneous cases. Although G:C to A:T transition and G:C to T:A transversion were commonly observed in MGT-induced and spontaneous groups, specific MFs of both types of mutations were significantly increased in MGT instilled animals.

**Table 2:** Classification of *gpt* mutations isolated from the lungs of control and MGT-treated mice.

Type of mutation			Control		MGT		*P* value *
			Number of mutants (%)	Specific MF ^†^ (×10^−6^)	Number of mutants (%)	Specific MF ^†^ (×10^−6^)	
Base substitution	Transition	G:C to A:T	8 (29.6)	0.89	14 (46.7)	4.71	0.00002
		A:T to G:C	4 (14.8)	0.44	2 (6.7)	0.67	0.62571
	Transversion	G:C to T:A	6 (22.2)	0.66	6 (20)	2.02	0.04258
		G:C to C:G	0 (0)	0.00	0 (0)	0.00	-
		A:T to T:A	0 (0)	0.00	0 (0)	0.00	-
		A:T to C:G	2 (7.4)	0.22	2 (6.7)	0.67	0.2417
Insertion			2 (7.4)	0.22	1 (3.3)	0.34	0.73017
Deletion			5 (18.5)	0.55	4 (13.3)	1.35	0.17072
Others			0 (0)	0.00	1 (3.3)	0.34	0.08114
Total			27(100)	-	30 (100)	-	-

^†^: Specific MF was calculated by multiplying the total mutation frequency by the ratio of each type of mutation to the total mutation; and *: *P* values were determined using Fisher’s exact test according to Carr and Gorelick [20].

### 2.4. Microscopic Findings in the Lungs of gpt Delta Transgenic Mice Administered MGT

**Figure 4:**
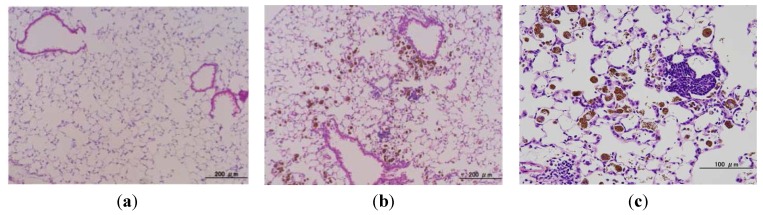
Microscopic findings in the lungs of *gpt* delta mice intratracheally instilled with MGT.Representative histopathology of the lungs of: (**a**) a control mouse given vehicle (once a week for 4 weeks; killed at 22 weeks of age); and (**b**,**c**) a mouse given multiple doses of 0.2 mg MGT (killed at 22 weeks of age). The brown-colored material is MGT.

There were no obvious histopathological changes in the lungs of vehicle-treated control mice (Figure 4a). In mice given multiple MGT administrations (0.2 mg weekly for 4 weeks), macrophage phagocytising MGT were recruited diffusely in the alveolar lumina and occasionally also in the alveolar interstitium (Figure 4b,c). Inflammatory cell infiltration (mainly lymphocytes) occurred in the alveolar interstitium and around vascular vessels or bronchioles, with granulations being scattered (Figure 4b,c). In addition, swelling of Type II alveolar epithelial cells and hyperplasia of bronchial epithelial cells were also observed (Figure 4b,c). Similar findings, but with a smaller degree of particle accumulation and granuloma formation, were observed in the lungs of mice that received a low-dose MGT instillation (data not shown).

### 2.5. Quantification of Oxidative and Lipid Peroxide-Related DNA Adducts

**Figure 5:**
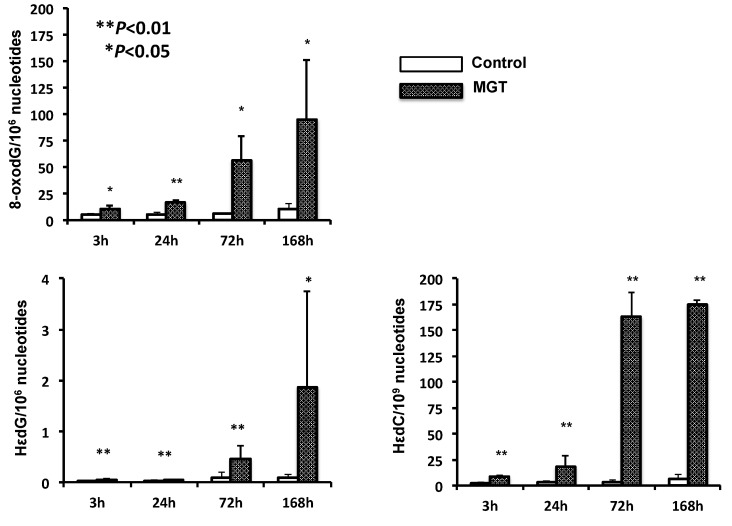
Oxidative and lipid peroxide-related DNA adduct formation induced by MGT exposure in the lungs of ICR mice. DNA was extracted from the lungs 3, 24, 72 and 168 h after intratracheal instillation of 0.2 mg of MGT, and was digested enzymatically. Control samples were obtained from the lungs of mice given the vehicle for the same durations of MGT exposure. 8-Oxo-7,8-dihydro-2′-deoxyguanosine (8-oxodG) and two types of Hε-adduct were quantified by stable isotope dilution liquid chromatography-mass spectrometry (LC-MS/MS). Asterisks (* and **) indicate a significant difference (*P* < 0.05 and *P* < 0.01) from vehicle control (treatment with 0.05% (*v*/*v*) Tween-80) at the same point in the Student’s *t*-test.

Levels of the DNA adduct analyzed in lung DNA extracted from MGT-treated mice (0.2 mg per capita) at 3, 24, 72 and 168 h after exposure are shown in Figure 5. DNA adducts related to oxidative stress and lipid peroxidation (8-oxodG, HεdC and HεdG) were all increased up to 168 h. 8-oxodG adduct was more abundant than those of HεdC and HεdG.

## 3. Discussion

The present study showed MGT to clearly exert genotoxicity in the lungs of mice when using a comet assay. We only analyzed a single time point (3 h) in the present study, however the levels of oxidative- and inflammation related-DNA adducts gradually increased in a time dependent manner (Figure 5). Thus, it is suggested that DNA damage induced by MGT instillation might chronically continue for a certain period. In addition to the *in vivo* genotoxicity, we showed MGT to be mutagenic in the lungs of mice, using *gpt* delta transgenic mice. To further elucidate the mechanisms behind the increase in MF observed in this study, we analyzed mutation spectra using a PCR-direct sequencing method. Most mutations induced by MGT in the present study occurred at G:C base pairs (66%). The prominent mutation types induced by MGT were a G:C to A:T transition followed by a G:C to T:A transversion. In contrast, G:C to C:G transversion was not more frequent with MGT treatment, whereas this type of transversion is commonly increased by fullerene, kaolin and multi-walled carbon nanotubes (MWCNTs) [21,22,23]. From this observation, it is suggested that mechanisms leading to the induction of mutations in mice lungs by MGT might be somehow different from other particles, such as fullerene, kaolin and MWCNTs. In general, the G:C to A:T transition and G:C to T:A transversion have commonly been observed in spontaneous mutants. It has been reported that deamination of 5-methylcytosine might be involved in G:C to A:T transitions occurring at the 5′-CpG-3′ site [24,25]. Even though G:C to A:T transitions were increased by MGT exposure, the percentage of such substitutions occurring at the 5′-CpG-3′ site slightly decreased compared to the vehicle control (7/14 = 50% for MGT treated mice *versus* 6/8 = 75% for control mice, data not shown), thus it is likely that MGT might increase G:C to A:T transitions other than deamination of 5-methylcytosine.

To examine the mechanisms of mutation induced by MGT, we further performed oxidative and lipid peroxide-related DNA adduct analysis. Levels of 8-oxodG were significantly increased in MGT-treated mice, and the level was maintained for a week. Similarly, the levels of HεdC and HεdG also increased in the lungs of mice with MGT-exposure. These DNA adducts are derived from lipid peroxidation, suggesting that MGT may induce oxygenation of lipids in tissues caused by generation of ROS via inflammatory responses. In general, 8-oxodG causes transversion mutations (G:C to T:A) in DNA because it can base pair with adenine as well as cytosine [26,27]. Recently, it has been reported that HεdC predominantly induces C to A or T mutations in human cells [28,29]. The most prominent mutation type induced by MGT was G:C to A:T and G:C to T:A. Therefore, it is likely that inflammatory responses might exist in the mechanisms behind the increase in mutations by MGT treatment. Supporting this hypothesis, inflammatory changes, such as the infiltration of macrophages phagocytising MGT, were diffusely introduced in the lungs of MGT-treated mice (Figure 4b,c).

Recently, innate immune activation through Nalp3 inflammasomes has been suggested to play an important role in the pulmonary fibrotic disorders of silicosis and asbestosis [30,31]. Therefore, it is suggested that MGT can activate in the same way as asbestos and silica. On the other hand, it has been reported that Fe^2+^ ions could be released from MGT [32]. The free Fe^2+^ ions lead to damage of DNA via ROS generation. In fact, we have recently reported that MGT actually manifests ROS generation and then leads to cytotoxicity and clastogenicity in cultured mammalian cells [17,18]. Therefore, it is suggested that iron ions may partly contribute to the genotoxicity induced by MGT in the present study. In contrast, it has not been ruled out that direct interaction between MGT and biomolecules, such as protein and DNA, might be partly involved in induction of *in vivo* genotoxicity. Moreover, supermagnetic iron oxide nanoparticles composed of Fe_3_O_4_ binding histidine-rich proteins in physiological conditions has also been reported [33].

As mentioned above, MGT has been widely utilized in various fields, including diagnosis and drug-delivery [1,2,3,4]. Due to the biomedical applications, MGT must have multifunctional characteristics, such as optimized size and surface modifications. Recent data have shown that modification with different functional groups (–OH, –COOH, –NH_3_, *etc.*) and different sizes of MGT are critical determinants for various biological functions, including cellular responses, cytotoxicity and genotoxicity [34]. For example, magnetic albumin nanospheres, designed for drug delivery systems, showed neither toxicity nor alteration on histopathological examination [35]. Although various types of MGT have already been commercialized for clinical use, their toxicity has not been fully elucidated yet. Therefore, studies for the genotoxicity of modified MGT are also needed. Moreover, to improve their safety for clinical application, it is necessary to clarify the mechanisms for genotoxicity of modified MGT.

## 4. Experimental Section

### 4.1. Materials and Chemicals

MGT was purchased from Toda Industrial Co. Ltd. (Hiroshima, Japan). MGT without any chemical modifications or coatings was used in the present study. The characteristics of MGT were the following (data sheet by the Toda Kogyo Corporation (Hiroshima, Japan) and reference): spherical shape; an average particle size of 10 nm measured by transmission electron microscopy (TEM); a size of 60–100 nm as measured by dynamic light scattering (DLS); a zeta potential of 30–40 mV at pH 10; and a surface area in powder of 100–120 m^2^/g.

2′-Deoxyguanosine was obtained from Tokyo Kasei (Tokyo, Japan). l(+)-ascorbic acid, NucleaseP1, dimethyl sulfoxide (DMSO) and high performance liquid chromatography (HPLC) grade methanol were purchased from Wako (Tokyo, Japan). Phosphodiesterase I was purchased from Worthington Biochemical Corp. (Lakewood, NJ, USA). Bovine spleen phosphodiesterase II, DNase I, Type I agarose, low melting point agarose, and Triton X-100 and Bacterial alkaline phosphatase Type III (*E. coli*) were purchased from Sigma Co. (St. Louis, MO, USA). Ethidium bromide was obtained from Merck (Darmstadt, Germany). All other chemicals used were of analytical grade and purchased from Wako.

### 4.2. Animals

Male imprinting control region (ICR) mice (six weeks old) and *gpt* delta mice (nine weeks old) were obtained from Japan SLC (Shizuoka, Japan). The *gpt* delta mice carry approximately 80 copies of lambda EG10 DNA on each chromosome 17 on a C57BL/6J background [20]. Animals were provided with food (CE-2 pellet diet, CLEA Japan, Inc., Tokyo, Japan) and tap water *ad libitum* and quarantined for one week. Mice were maintained under controlled conditions: twelve-hour light/dark cycle, 22 ± 2 °C room temperature, and 55% ± 10% relative humidity. The experiments were conducted according to the “Guidelines for Animal Experiments in the National Cancer Center” of the Committee for Ethics of Animal Experimentation of the National Cancer Center.

### 4.3. Preparation and Characterization of MGT

MGT particles were diluted in water at a concentration of 0.5 mg/mL or 2.0 mg/mL and suspended by sonication for 10 min before intratracheal administration for each test. Physical characterization of particles including crystal appearance and diameters of nanoparticles were observed and measured under a scanning electron microscope (SEM). The size distribution of materials used in the present study was analyzed by DLS. Zeta potential of MGT was measured using ZetasizerNano (Malvern Instruments Ltd., Malvern, UK).

### 4.4. Comet Assay

For the comet assay, five male ICR mice were intratracheally instilled with particles using a polyethylene tube under anesthesia with 4% halothane (Takeda Chemical, Osaka, Japan). Single doses of 0.05 mg or 0.2 mg per animal were employed. The control mice (*n* = 5) were instilled intratracheally with 0.1 mL of the solvent alone. The mice were sacrificed 3 h after these particle administrations, and lungs were then removed and used for comet assay immediately. The alkaline comet assay was performed according to the method of our previous paper [21].

### 4.5. gpt and Spi− Mutation Assay

For mutation analysis, each group of five to six male *gpt* delta mice was intratracheally instilled with particles at multiple doses of 0.05 mg or 0.2 mg per animal per week for four consecutive instillations. The control mice (*n* = 5) were instilled intratracheally with the solvent alone. The mice were sacrificed at 22 weeks of age, *i.e.*, 8 weeks after particle administrations, and the lungs were stored at −80 °C until the DNA was isolated. High-molecular-weight genomic DNA was extracted from the lungs using a RecoverEase DNA Isolation Kit (Stratagene, La Jolla, CA, USA) according to the supplier’s instructions. Lambda EG10 phages were rescued using Transpack Packaging Extract (Stratagene). The *gpt* mutagenesis assay was performed according to previously described methods [21,22,23,36].

### 4.6. Histopathological Evaluation

For histopathological evaluation, lungs obtained from *gpt* delta mice with or without nanoparticle instillation (*n* = 2 or 3) were fixed in 10% neutral buffered formalin, embedded in paraffin blocks, and routinely processed to hematoxylin and eosin-stained sections.

### 4.7. Oxidative and Lipid Peroxide-Related DNA Adduct Formation

For DNA adduct analyses, each group of five male ICR mice was intratracheally instilled with MGT at a single dose of 0.2 mg per animal, and sacrificed 3, 24, 72 or 168 h after nanoparticle administration. Control samples were obtained from the lungs of mice given the vehicle. Mouse lung DNA was extracted and purified using a Gentra^®^ Puregene™ tissue kit (QIAGEN, Valencia, CA, USA). The protocol was performed according to the manufacturer’s instructions except that desferroxamine (final concentration: 0.1 mM) was added to all solutions to avoid the formation of oxidative adducts during the purification step. The extracted DNA was stored at −80 °C until analysis for DNA adducts. Mouse lung DNA (40 μg) extracted from vehicle (*n* = 5) and MGT treated (*n* = 5) mice DNA were enzymatically digested, and 8-oxodG, Hε-dG and Hε-dC were analyzed and quantified using the same procedure previously described [22].

### 4.8. Statistical Analysis

The data obtained from the comet assay are expressed as mean ± standard errors. The data from *gpt* and Spi^−^ mutation assay are expressed as mean ± standard deviations. To test for significant differences of tail moment in the comet assay between a group treated with materials and an untreated group, Dunnett’s test after one-way ANOVA was used to evaluate the differences; *P* values lower than 0.05 were considered to indicate statistical significance. The data were statistically compared with the corresponding solvent control using the Student’s *t* test for mutation assay and DNA adduct formations. The data were compared with the corresponding solvent control using the *F* test before application of the Student’s *t* test. If the *F* test evaluation showed an unequal variance, the *P* value was determined using the Welch’s *t* test. In the case of the mutation spectrum analysis, *P* values were determined using Fisher’s exact test according to Carr and Gorelick [20]. *P* values lower than 0.05 were considered to indicate statistical significance.

## 5. Conclusions

We have clearly demonstrated that MGT induce genotoxicity in intratracheally instilled mouse lungs. Based on the mutation spectrum, histopathological evaluation, and DNA adducts analyses, it is suggested that inflammatory responses lead to oxidative- and lipid peroxide-related DNA adduct formations, and this might contribute to the genotoxicity induced by MGT treatment. The mechanisms are not fully understood yet, and further studies of the mechanisms of genotoxicity are needed.
